# Supplementary material for: Multiple Early Life Stressors as Risk Factors for Neurodevelopmental Abnormalities in the F1 Wistar Rats
Source: Brain Sci. 2023 Sep 22;13(10):1360. doi: 10.3390/brainsci13101360 (PMC10605318; doi:10.3390/brainsci13101360)
Supplement: Supplementary file 1 [file brainsci-13-01360-s001.zip › brainsci-2520949-supplementary.pdf]

Table S1. Detailed composition of low protein (LP; 8% protein) and normal control (NC; 20% protein) experimental diets.

S. No.	Constituents of Diet	8% protein (LP)	20% protein (NC)
1.	Wheat	56.20%	22.50%
2.	Bengal gram	0	60.00%
3.	Groundnut oil	3.00%	4.00%
4.	Soyabean oil	3.00%	0
5.	Casein	0	4.00%
6.	Skimmed milk powder	6.00%	5.00%
7.	Vitamin mixture	0.50%	0.50%
8.	Mineral mixture	0.50%	0.40%
9.	Starch	23.90%	0
10.	Sodium chloride	0.30%	0
11.	Di calcium phosphate	0.40%	0
12.	Calcium carbonate	0.90%	0
13.	Choline chloride	0.20%	0
14.	L-Cysteine	0.10%	0
